# Coordinated Fc-effector and neutralization functions in HIV-infected children define a window of opportunity for HIV vaccination

**DOI:** 10.1097/QAD.0000000000002976

**Published:** 2021-06-10

**Authors:** Eunice W. Nduati, Mathew J. Gorman, Yiakon Sein, Tandile Hermanus, Dansu Yuan, Ian Oyaro, Daniel M. Muema, Thumbi Ndung’u, Galit Alter, Penny L. Moore

**Affiliations:** a KEMRI Wellcome Trust Research Programme, Kilifi, Kenya; b Ragon Institute of MGH, MIT and Harvard, Cambridge, MA, USA; c National Institute for Communicable Diseases of the National Health Laboratory Services, Johannesburg; d Africa Health Research Institute, Durban; e HIV Pathogenesis Programme, The Doris Duke Medical Research Institute, University of KwaZulu-Natal, Durban, South Africa; f Max Planck Institute for Infection Biology, Berlin, Germany; g Division of Infection and Immunity, University College London, London, UK; h Antibody Immunity Research Unit, University of the Witwatersrand, Johannesburg, South Africa.

**Keywords:** antibodies, children, Fc-mediated function, HIV, neutralizing function, vaccines

## Abstract

**Objectives:**

Antibody function has been extensively studied in HIV-infected adults but is relatively understudied in children. Emerging data suggests enhanced development of broadly neutralizing antibodies (bNAbs) in children but Fc effector functions in this group are less well defined. Here, we profiled overall antibody function in HIV-infected children.

**Design:**

Plasma samples from a cross-sectional study of 50 antiretroviral therapy-naive children (aged 1–11 years) vertically infected with HIV-1 clade A were screened for HIV-specific binding antibody levels and neutralizing and Fc-mediated functions.

**Methods:**

Neutralization breadth was determined against a globally representative panel of 12 viruses. HIV-specific antibody levels were determined using a multiplex assay. Fc-mediated antibody functions measured were antibody-dependent: cellular phagocytosis (ADCP); neutrophil phagocytosis (ADNP); complement deposition (ADCD) and natural killer function (ADNK).

**Results:**

All children had HIV gp120-specific antibodies, largely of the IgG_1_ subtype. Fifty-four percent of the children exhibited more than 50% neutralization breadth, with older children showing significantly broader neutralization activity. Apart from ADCC, observed only in 16% children, other Fc-mediated functions were common (>58% children). Neutralization breadth correlated with Fc-mediated functions suggesting shared determinants of enhanced antibody function exist.

**Conclusions:**

These results are consistent with previous observations that children may develop high levels of neutralization breadth. Furthermore, the striking association between neutralization breadth and Fc effector function suggests that HIV vaccination in children could yield multifunctional antibodies. Paediatric populations may therefore provide an ideal window of opportunity for HIV vaccination strategies.

## Introduction

Broadly neutralizing antibodies (bNAbs) to HIV-1 are of particular interest for vaccine-mediated humoral immunity. Studies in macaque models [1–4], humanized mice [5] and humans [6] have provided proof-of-principle that a vaccine capable of inducing these types of antibodies is likely to be effective against most circulating HIV strains. However, only a small proportion of HIV-infected individuals generate bNAbs [7] and no HIV vaccine candidate has been able to elicit antibodies with sufficient breadth [8,9].

Antibody functions have largely been described in adults but less is known for children despite their distinct course of HIV infection. AIDS typically develops more rapidly in paediatric HIV cases compared with adults [10–12] and infants and children rapidly develop broader and more potent neutralizing antibodies than adults [13–15]. Furthermore, an isolated bNAb from an infant showed low somatic hypermutation and lacked insertions and deletions typical of bNAbs from adults, suggesting that infants may have a more direct pathway to breadth that does not require years of affinity maturation [13].

Antibodies are, however, multifunctional and their antiviral activity results from the synergistic functions of the fragment antigen binding (Fab) and fragment crystallizable (Fc) regions [16,17]. Antibodies capable of mediating Fc-effector functions via innate immune cells are commonly identified in HIV-infected people, and may contribute to viral control [18,19] and to slowing HIV acquisition [20]. Additionally, elite controllers may have more Fc-mediated antibody polyfunctionality that recruits a more coordinated innate immune response [21]. In the only HIV vaccine trial that has shown any protection to date, lower risk of HIV acquisition was associated with Fc-mediated antibody functions [22]. Fc-mediated functions also contribute to optimal antiviral activity for some bNAbs [23]. BNAbs were shown to interfere with the establishment of a silent reservoir through Fc-Fc receptor-mediated mechanisms [24]. Furthermore, reduced protection by passively administered bNAbs occurred when Fc-receptor activity was engineered out of these antibodies [25]. In infected adults, HIV-specific Fc-effector functions early in HIV infection predicted the downstream development of bNAbs [26] but such studies have not been performed in children.

Understanding the development of broadly neutralizing and Fc-mediated antibody functions in paediatric natural HIV infection remains important. To address this, we assessed the presence, magnitude and correlation of neutralizing and Fc-mediated functions in a cross-sectional study of 50 antiretroviral therapy (ART)-naive, chronically infected children aged 1–11 years, predominantly infected with HIV clade A. Fifty-four percent of the children developed neutralization breadth against the panel of viruses used in this study. There was a significant association of antibody neutralization breadth with the Fc-mediated functions suggesting common determinants of function. These results agree with the accumulating findings that children may have unique immunological profiles that favour the development of more effective antibodies and understanding of these profiles may inform vaccine strategy.

## Materials and methods

### Ethics statement

The study used samples from a previously reported parent study [27]. Ethical approval was received from the Kenya Medical Research Institute Science and Ethics Review Unit (SERU-3530). Informed consent for study participation and sample storage was obtained from each child's parent/guardian.

### Study population

Recruitment of study participants is reported elsewhere [27]. In summary, HIV-infected children aged 1–11 years were recruited into a cross-sectional study between October 2010 and May 2012 during care or first visit to the Kilifi County Hospital, in Coastal Kenya, where HIV-1 clade A is predominant. At the time of recruitment, the WHO's treatment guidelines recommended: all those less than 24 months: combination ART (cART); 25–59 months: cART if CD4^+^ T-cell percent less than 25% and/or if in WHO clinical stage 3 or 4; greater than 60 months: cART if their CD4^+^ T-cell percent less than 20% and/or if in WHO clinical stage 3 or 4 [28]. Of the 121 children invited for study participation, guardians/parents of 50 children self-reported that the child had not initiated ART, and these children were included in this study.

### Determination of HIV-specific antibody neutralization breadth

Antibody neutralization was determined against a globally representative panel of 12 viruses belonging to subtypes A (*n* = 1), B (*n* = 2), C (*n* = 3), G (*n* = 1) and circulating recombinant forms (CRFs) (*n* = 5) [15,29]. Neutralization was measured as previously described [30–33] by a reduction in luciferase gene expression after single-round infection of TZM-bl cells with Env-pseudotyped viruses. Antibody potency was calculated as the plasma dilution needed to neutralize 50% viral infectivity, and breadth as the ability to neutralize more than 50% of this multisubtype panel.

### Determination of HIV-specific antibody subclass levels

#### Multiplex assay

A previously described multiplex assay was adapted [34], whereby microsphere beads were coupled to HIV antigens clade A Q461.D1 gp120, clade A BG505 SOSIP, clade A 92RW020 gp140, clade A 94UG103 gp120, clade B JRFL gp140, clade B MN gp120, clade C TV-1 gp140 and clade C IAVIC22 gp120, and two control antigens HA (A/California/07/2009), HA (A/Victoria/3/75) and inactivated Tetanus toxoid. Coupled beads were incubated with diluted plasma overnight at 4 °C and the levels of total IgG and IgG_1_, IgG_2_, IgG_3_, IgG_4,_ IgA_1_ and IgA_2_ detected by PE-conjugated detection agents (Southern Biotech, Birmingham, Alabama, USA) using an IQue Screener Plus (IntelliCyt).

#### ELISA

In brief, 50 μg of the recombinant BG505 clade A gp120 (donated by Elise Landais) were used to coat plates prior to incubation with diluted test plasma. Subclass-specific antihuman immunoglobulin conjugated to alkaline phosphatase (mouse anti-IgG, anti-IgG_1_, anti-IgG_2_, anti-IgG_3_, anti-IgG_4_ or goat anti-IgA) from Southern Biotech, USA were used for antibody subclass detection. Relative arbitrary antibody units were calculated by interpolating OD readings to standard curves prepared using pooled hyper-reactive HIV-positive plasma.

### Determination of HIV-specific Fc-mediated antibody functions

#### Antibody-dependent phagocytosis

Fluorescent neutravidin yellow-green beads (Invitrogen, Waltham, Massachusetts, USA) were coated with AVI-tag biotinylated clade A gp120 (donated by Devin Sok and Joseph Jardine). The ability of patient plasma samples to generate antigen-specific immune-complexes that a) drive their uptake by a monocytic cell line (THP-1; ATCC TIB-202), to establish antibody-dependent cellular phagocytosis (ADCP), b) drive their uptake by neutrophils enriched from healthy donor's whole blood, to establish antibody-dependent neutrophil phagocytosis (ADNP), measured by flow cytometry [21,35,36]. Neutrophils were defined as CD3−CD14−CD66b+ cells (antihuman CD3 AF700 and CD14 APC-Cy7 (BD Biosciences, San Jose, California, USA) and antihuman CD66b Pacific Blue (BioLegend, San Diego, California, USA)], whereas THP-1 cells were left untreated. The phagocytic activity was presented as a score calculated as the [(% cells that have taken up antigen-coupled beads) × (MFI (mean fluorescent intensity) of cells that have taken up antigen-coupled beads)]/10000.

#### Antibody-dependent complement deposition

Fluorescent neutravidin red beads (Invitrogen) were coupled with AVI-tag biotinylated clade A gp120 and then incubated with diluted patient plasma, prior to adding low-tox guinea pig complement (Cedarlane, Burlington, North Carolina, USA). Antibody activation of the complement resulted in release of C3, which was measured using antihuman C3 FITC goat IgG (BD Biosciences) by flow cytometry.

#### Antibody-dependent natural killer cell degranulation

In brief, plates were coated with AVI-tag gp120, blocked, and patient plasma added before incubating for 2 h at 37 °C. NK cells isolated from a healthy donor's whole blood were added simultaneously with anti-CD107a PE-Cy5 (BD Biosciences), Brefeldin A (Sigma) and Golgi Stop (BD Biosciences) and incubated for 5 h at 37 °C as previously reported [37]. NK cells were then stained with anti-CD56 PE-Cy7, anti-CD16 allophycocyanin (APC)-Cy7 and anti-CD3 Alexa Fluoro 700 (BD Biosciences), fixed (FIX&PERM cell fixation and permeabilization kit, Thermo Fisher Scientific, Waltham, Massachusetts, USA), and stained intracellularly with anti-IFNγ-APC and anti-MIP-1β-PE (BD Biosciences). Surface expression of CD107a and intracellular production of IFNγ and MIP1β by NK cells (CD16+/56+CD3−) were then analysed by flow cytometry. For each Fc-assay, a negative control (human purified IgG, Sigma) was included. Mean of signal in the negative controls wells was subtracted as background signal. The gating strategy for the Fc-mediated functions are illustrated in Supplementary Fig. 1.

### Fc-mediated polyfunctionality

Polyfunctionality is represented as the summed *z* scores for each child's Fc-mediated function. *z* scores were determined by subtracting the mean function of all children from an individual child's value and dividing this by the standard deviation.

### Analysis

Statistical analyses were performed in Graphpad Prism version 8.4.1 (GraphPad, Software, San Diego, California, USA). Mann--Whitney nonparametric tests were used to compare groups and associations determined by Spearman test. *P* less than 0.05 considered significant.

## Results

### Study population clinical characteristics

Children were categorized by age, as per the ART initiation WHO guidelines at the time of study [28] (Table 1). Five children had age data missing from the records, four were less than 24 months of age, 17 were between 25 and 59 months, and 24 were above 59 months of age. Forty-six percent (23 of 50) of the children were female (Table 1). Viral load data was available for 44 of 50 with a medium HIV RNA copies/ml of 36 644 [IQR, 1815–98565], median CD4^+^ cell counts/μl blood was 912 [IQR, 608–1422], median percentage CD4^+^ was 20.8 [IQR, 13.0–29.0], lymphocyte counts 4.0 [3.6–5.5] × 10^3^ cells/μl, whereas the median haemoglobin levels was 10.0 [10–11] mg/dl for all the children.

**Table 1:** Clinical characteristics of the study population at the time of sampling.

Clinical parameters		*N* or median [25th–75th percentile]
Age (years)	Missing	5
	<24 months	4
	25–59 months	17
	60 months	24
Gender	Female	23
	Male	27
% CD4^+^	<24 months	22.4 [14.6–30.2]
	25–59 months	20.7 [15.4–30.9]
	60 months	20.8 [9.3–25.9]
	All children	20.8 [13.0–28.8]
CD4^+^ cell count (cells/μl)	<24 months	1979 [1201–2739]
	25–59 months	1034 [759–1580]
	60 months	715 [567–1052]
	All children	912 [608–1422]
Viral load (copies/ml)	<24 months	1314.5 [913.5–272 752]
	25–59 months	37 075 [2687–123 335]
	60 months	36 749 [7632–92 559]
	All children	36 644 [1815–98 565]
Lymphocyte count (10^3^/μl)	<24 months	8.1 [6.4–12.5]
	25–59 months	5.0 [4.0–5.9]
	60 months	3.8 [3.4–4.3]
	All children	4.0 [3.6–5.5]
Hb conc. (mg/dl)	<24 months	10.0 [8–12]
	25–59 months	10.0 [9–11]
	60 months	10.5 [10–11]
	All children	10.0 [10–11]

Baseline characteristics for the children at enrolment into study. Children were divided into age brackets as per the WHO ART initiation guidelines at the time of study. Five children had age missing from the data records and age is indicated as missing. Lymphocyte counts are represented as counts per 10^3^ cells per uL of blood. All children; total number of children included in the study. *N*, total number of children; Hb, haemoglobulin.

### HIV-specific antibody neutralization activity

**Fig. 1:**
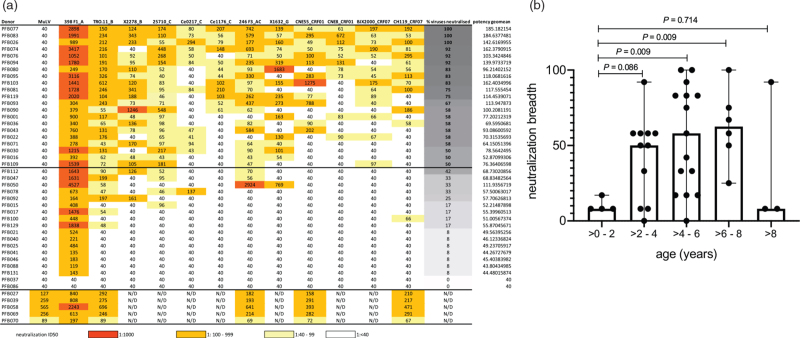
HIV-specific antibody neutralization function.

Neutralization breadth was determined for 44 children against a globally representative panel of 12 viruses (6 children were excluded as EDTA plasma was not available, and heparin plasma results in high background in this assay) (Fig. 1a). Of the 44 plasma samples tested, five showed activity against murine leukaemia virus (MuLV) envelope pseudotyped-viruses, a negative control, suggesting these plasmas contained antiretroviral drugs, despite self-reporting. These children were, therefore, excluded from all further analysis.

(a) Antibody neutralization was determined against 12 globally representative viruses using the TZM-bl neutralization assay. Neutralization titre is represented as the reciprocal of the plasma dilution required to inhibit 50% virus infection (ID_50_). Data shown as a heat map with titres greater than 1000 in red, 100–999 in orange, 41–99 in yellow and less than 40 in white. Titres of 1 : 40 or less indicate no neutralization. Neutralization breadth per sample; percentage of viruses neutralized. Potency; plasma dilution needed to achieve 50% viral infectivity and the geometric mean determined. (b) Neutralization breadth by age. Mann--Whitney test used and median and range indicated. *P* less than 0.05 considered significant.

Virus neutralization ranged from 0 to 100% breadth against this panel, with potency highest against the tier 1 virus 398F1, and moderate titres against the rest, which are tier 2 viruses, more typical of circulating strains (Fig. 1a). Fifty-four percent of the children (21 of 39) neutralized more than half of the viruses tested and were defined as broad neutralizers in this study, including three children who neutralized all the viruses in this panel. Neutralization breadth was significantly higher in the older children, plateauing at about 4 years after infection (Fig. 1b) similar to studies of neutralization breadth in adults [32,33]. Plasma neutralization breadth and geometric mean titre were linearly associated (Spearman's rho = 0.98, *P* ≤ 0.0001) (Supplementary Fig. 2a), and therefore, neutralization breadth was used in all downstream association analyses.

### HIV-specific antibody levels and subtypes

We next assessed HIV-specific antibody levels and isotype-specific binding to several HIV antigens. All children had IgG responses to the clade A BG505 gp120, as measured by ELISA. A large proportion of these were IgG_1_ with antibody levels to the other IgG subclasses or IgA being lower (Supplementary Fig. 3a).

**Fig. 2:**
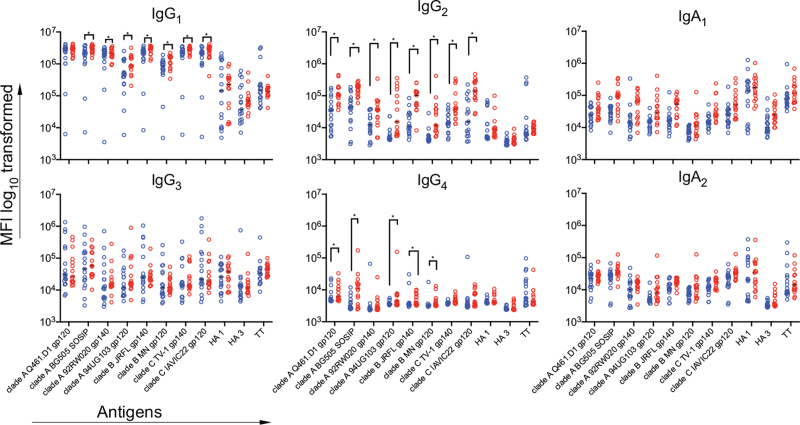
Comparison of HIV-specific antibody levels in children with and without antibody neutralizing breadth.

A similar pattern in antibody classes and isotype levels was observed using a multiplex microsphere bead assay incorporating a range of HIV antigens (Fig. 2). Antibody cross-reactivity to envelopes from clades B and C were observed and for some of the antigens, binding was at similar levels to the clade A antigen responses. Similar to the ELISA data, the predominant antibody subclass was IgG_1_. For IgG_1_, IgG_2_ and IgG_4_, binding levels to several antigens were higher in children who developed neutralization breadth. This was HIV-specific as antitetanus toxoid and anti-HA influenza antibodies (Fig. 2) and total IgG were similar in both groups (Supplementary Fig. 3b).

Antigen-specific IgG_1_, IgG_2_, IgG_3_, IgG_4_, IgA_1_ and IgA_2_ antibodies were quantified using an antigen-specific isotype multiplex assay. *x*-axis, antigens tested, *y*-axis log_10_ transformed mean fluorescence intensity (MFI). Blue open circles indicate children with less than 50% neutralization breadth; red open circles indicate children with greater than 50% neutralization breadth. The median of antibody levels against each antigen is presented. Nonparametric Mann--Whitney test with *P* less than 0.05 being significant used for comparison. Each sample was analysed in technical duplicates. H1, A/California/07/2009; H3, A/Victoria/3/75; TT; inactivated Tetanus toxoid.

### HIV-specific Fc-mediated antibody functions and polyfunctionality in children

**Fig. 3:**
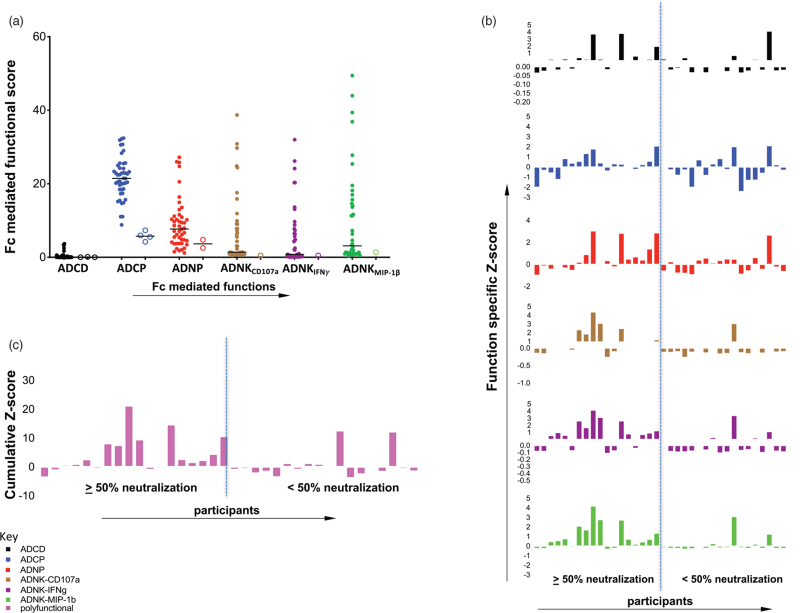
HIV-specific Fc-mediated antibody functions.

Fc-mediated functions varied across the participants and by function. Complement deposition was detected in only 16% (8 of 50) of children, as expected from the low IgM titres and IgG_3_ titres observed. A large proportion of the children's plasma, initiated phagocytosis either by a monocyte cell line THP-1 (ADCP) (100% of the children), or by neutrophil uptake (ADNP) (82% of the children) (Fig. 3a) with these two assays correlating (Spearman rho = 0.58, *P* < 0.0001) confirming similar mechanisms (Supplementary Fig. 4a) [38]. The expression of CD107a (a marker expressed upon natural killer cell degranulation) and IFNγ and MIP-1β, two intracellular cytokines produced upon activation, were measured as proxies of antibody-dependent natural killer (ADNK) cell function. These were variable across samples, with ADNK_CD107a_, ADNK_IFNγ_, ADNK_MIP-1β_ detected in 98, 58, and 60% of children, respectively. CD107a expression and IFNγ and MIP-1β production were directly correlated as previously observed [21,39] (Supplementary Fig. 4b--d). Therefore, either of these markers could be used independently as a marker for natural killer cell function.

The ability of diluted children plasma to drive the uptake of gp120-coated fluorescent beads by THP-1 cells [antibody-dependent cellular phagocytosis (ADCP)], by neutrophils [antibody-dependent neutrophil phagocytosis (ADNP)], promote complement deposition [antibody-dependent complement deposition (ADCD)]; or activate NK cells [antibody-dependent natural killer (NK) cell degranulation (ADNK)] reflected by surface expression of CD107a and IFNγ and MIP-1β production was evaluated. (a) Median functional score for the tested Fc-mediated functions. Median score shown, open circles; HIV-negative controls, closed circles; HIV-positive participants. (b) z scores for each child's Fc-mediated functions. (c) Polyfunctionality score (summed *z* scores of each Fc-mediated function for each child). Experiments were performed in duplicates.

*Z* scores for each Fc function were used to compare the distribution of functions between neutralizers and nonneutralizers. A positive *z* score was more common in children who developed neutralization breadth suggesting the possibility of common determinants for function (Fig. 3b). To establish if these independent Fc-mediated functions were coordinated, we calculated a polyfunctionality score. A summed *z* score greater than 0 represented coordinated Fc-mediated functions (Fig. 3c). Thirty-six children with a polyfunctionality score had neutralization data available. Notably, 13 of 18 of the children who generated neutralization breadth showed coordinated Fc-mediated antibody functions compared with five of 18 of the children with poor neutralization breadth. Our data show that the development of neutralization breadth was associated with a more coordinated Fc-mediated function.

### Association of antibody titres and functions with clinical parameters

Given the range of Fc effector functions and antibody titres in this cohort, we sought to understand the association of these with clinical parameters, such as age, CD4^+^ frequency (% of total PBMCs), CD4^+^ counts and viral load. As HIV infection in this cohort was a result of vertical transmission from mother to child, the child's age reflects duration of HIV infection.

**Fig. 4:**
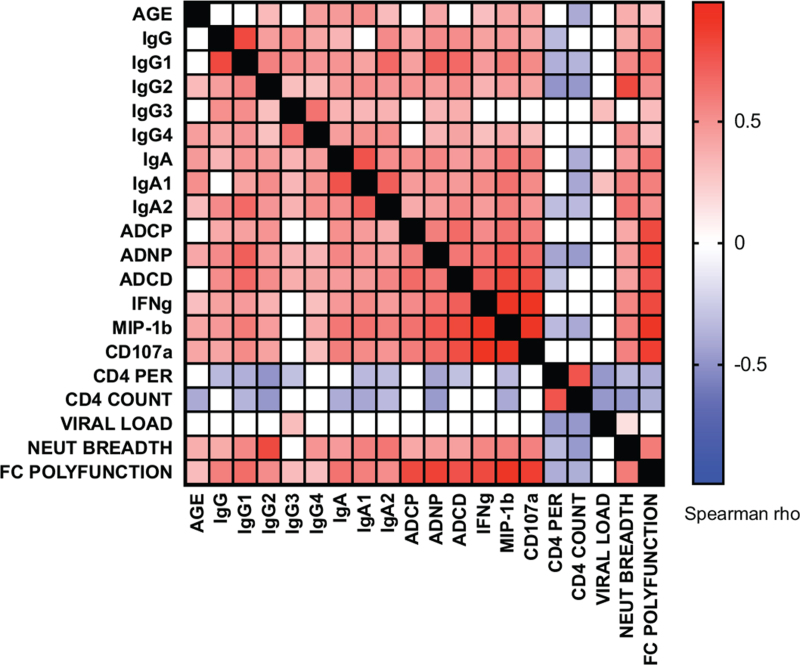
Heatmap representation of HIV-specific antibody function association with clinical outcomes, antibody levels and classes/subtypes: Spearman rho is shown where *P* less than 0.05, white boxes represent no significant association.

ADCD, antibody-dependent complement deposition; ADCP, antibody-dependent cellular phagocytosis; ADNK, antibody-dependent natural killer (NK) cell degranulation functions; ADNP, antibody-dependent neutrophil phagocytosis; NEUT BREADTH, neutralization breadth.

As the pattern of antibody levels was similar across the eight gp120 HIV antigens tested (Fig. 2), BG505 SOSIP responses, representative of the prevailing clade in the study region, were tested for their association with antibody function (Fig. 4). IgG antibody levels directly correlated with neutralization breadth (Spearman's rho = 0.3954, *P* = 0.0170) and with ADCD, ADCP, ADNP, ADNK_IFNγ_ and ADNK_MIP-1β_ secretion (Supplementary Table 1). Similarly, the coordinated Fc-mediated function represented by the polyfunctional score was associated with IgG titres (Spearman's rho = 0.5769, *P* = 0.0001).

IgG subclasses IgG_1_, IgG_2_ and IgG_4_ were also directly associated with antibody neutralization breadth (Supplementary Table 1, Spearman's rho = 0.5456, *P* = 0.0012; Spearman's rho = 0.8114, *P* = 0.0001; Spearman's rho = 0.4892, *P* = 0.0045, respectively) but IgG_3_ was not. Additionally, both IgG_1_ and IgG_2_ sub-classes showed strong associations with individual Fc-mediated functions, and with the overall polyfunctionality, whereas IgG_3_ and IgG_4_ were poorly associated. IgA antibody levels were also associated with neutralization breadth (Spearman's rho = 0.4621, *P* = 0.0046) and this association was observed for both the IgA_1_ and IgA_2_ subclasses. Similarly, IgA antibody levels were correlated with all Fc-mediated functions tested, and a strong association with Fc-mediated polyfunctionality score was also observed (Spearman's rho = 0.6313, *P* = 0.0001). Overall, higher antibody levels reflected better HIV-specific antibody functions.

As suggested in Fig. 1b, there was a direct association between age and neutralization breadth (Supplementary Table 1, Spearman's rho = 0.3766, *P* = 0.0258). Some Fc-mediated functions, namely ADNP and ADNK, also showed an association with age, suggesting these antibody functions improved over time. However, ADCP and ADCD were not associated with age, suggesting these Fc-mediated functions are generated early in infection and do not significantly improve over time.

Neutralization breadth was negatively associated with both CD4^+^ cell counts and percentage as in previous studies [15] (Supplementary Table 1, Spearman's rho = −0.4663, *P* = 0.0054, Spearman's rho = −0.3454, *P* = 0.0490, respectively). However, Fc-mediated functions were less strongly associated with reduced CD4^+^ cell counts and percentages. Although a consistent trend was observed, significance was only achieved for ADNP and ADNK_MIP-1β_ expression (Spearman's rho = -0.4582, *P* = 0.0020; Spearman's rho = -0.4120, *P* = 0.0074) and for the polyfunctional score (Spearman's rho = −0.3821, *P* = 0.0115). HIV-specific antibody functions were, therefore, not associated with slowed clinical deterioration. There was no significant association between viremia and neutralization breadth (Spearman's rho = 0.1509, *P* = 0.3870) or the different Fc-mediated antibody functions.

Taken together, these data suggest that neutralizers have greater, broader, and more functional humoral responses, which are in some cases correlated with age and CD4^+^ cell count, but independent of viral load.

## Discussion

Here, we measured HIV-specific antibody levels, neutralization breadth and Fc-mediated functions in chronically HIV-infected, ART-naive children. Neutralization breadth developed in more than half of the children and correlated with Fc-mediated functions, suggesting shared determinants of enhanced antibody function exist. Our observations support children's ability to recognize and mount robust immune responses against HIV antigens, confirming the potential of this target population for HIV immunization strategies.

Emerging data suggests that a larger proportion of children than adults develop neutralization breadth [14,15,40]. In agreement with this, we observed that 54% of the children neutralized more than 50% of the viruses in the representative test panel. Extended viral exposure may have allowed for activation of multiple B-cell lineages, favouring the acquisition of neutralization breadth. Additionally, higher numbers of effector T-follicular helper cells in children [41] may provide enhanced help than in adults for B-cell differentiation.

In this cross-sectional study, viral load was not an independent predictor of neutralization breadth. It is likely that the quality of antibody function at the time of sampling was determined by earlier or accumulated events over the course of infection, such as early viremia, as has been previously demonstrated [33]. The modest association previously reported [15] further supports the existence of alternative drivers of breadth in addition to viraemia.

Antibody neutralization breadth correlated with total IgG, IgG_2_ and IgA titres. Although IgG_2_ responses were lower, as previously reported [42,43], a strong association with neutralization breadth was observed, similar to adults [26]. Low peripheral IgA antibody levels were observed, in agreement with expected dominance in the mucosal environment [44,45]. However, these were highly associated with neutralization. It is likely that multiple Fc-related mechanism are involved in viral control [46,47] and that a balance in subtype and class distribution is necessary for the generation of antibody neutralization breadth [47]. Further studies to understand this linkage are necessary.

Only a small proportion of children had plasma antibodies that could drive complement deposition (ADCD). The undetectable levels of IgM (data not shown) and low IgG_3_ antibody levels, both good mediators of complement, may have contributed to low complement deposition. It is possible that after years of infection, circulating HIV-specific antibodies in these children were largely switched to IgG subtype [45]. IgM, a first-line antibody may have been detected earlier in infection and not at this chronic phase [48]. However, in the few children where ADCD was detectable, there was a strong association between complement deposition and total IgG and IgA as previously observed [26,49] and with IgG_1_. Higher IgG levels may have resulted in more IgG--antigen complex formation. Similarly, ADCD showed strong association with neutralization breadth supporting previous reports [26]. A potential mechanism for this association is the binding of C3 to complement-receptor-2 on follicular dendritic cells, which may improve antigen presentation in the germinal centres leading to higher affinity maturation and improved antibody breadth [50,51].

Unlike ADCD, the other Fc-mediated functions were more common. All the children in this cohort mediated ADCP, and the phagocytic score was high for most children as has been observed in adults over the course of HIV infection [26]. In RV144 vaccinees, depletion of IgG_3_ antibodies led to a significant loss of ADCP, although other subclasses were shown to be involved [52]. In our chronic infection cohort, few children had detectable levels of IgG_3_. Decreasing levels of IgG_3_ with disease progression have been previously reported [45,53,54] and may reflect downstream antibody switching. It is likely that the ADCP antibody function we observed was driven by highly functional IgG_1_ antibodies [55,56]. We further assayed phagocytosis via neutrophils (ADNP). Neutrophils are common and active in all sites of HIV infection. Phagocytosis by neutrophils has been reported to peak much faster than primary NK cell or monocyte-mediated responses suggesting its role in early viral control [57]. The induction of phagocytosis via distinct innate Fc effector cell types would be beneficial in mounting efficient viral clearance mechanisms.

We then measured multiple effector functions of activated NK cells including degranulation and the release of IFNγ and MIP-1β cytokines as proxies of antibody-dependent cell death. Previous comparisons have shown association between ADCC to CD107a degranulation [58] and in our study, there was positive correlation between CD107a, IFNγ and MIP-1β as previously observed [21,39] suggesting that either of these markers could be used as a proxy for ADCC. IgG_1_ mediates antiviral functions by binding to FcR, mediating ADCC of infected cells [59] and may have been critical in mediating the effector functions.

Previous studies suggest that multiple Fc-mediated functions provide superior viral clearance mechanisms by recruiting multiple players of innate immunity [21,26,37]. Here, 23 out of 50 (46%) children generated coordinated Fc-mediated functions. Importantly, a larger proportion of children who showed coordinated Fc-mediated effector functions also generated neutralization breadth, suggesting that antibodies function synergistically, consistent with emerging data of joint regulation of Fc-mediated and Fab-mediated antibody functions [26,60,61]. The identification of signatures for germinal centre activities that link these processes may be exploited in vaccine design to drive desired multifunctional antibody responses.

One limitation of the study is that IgG was not purified prior to performing the assays, which would have enabled a more realistic comparison of antibody ‘quality’. The cross-sectional study design also limited the association with earlier events, such as viral set point and changes over time or with clinical outcome.

Our findings show that children mount potent antibody responses to HIV antigens and that a large proportion develop neutralization breadth and Fc-mediated antibody functions. HIV vaccination strategies targeting children may, therefore, prove more likely to yield desired responses. The use of a coordinated multifunctional systems approach in key populations, such as paediatric donors, thus provides a comprehensive characterization of desirable antibody functions.

## Acknowledgements

We thank the study participants, the field workers, hospital staff for their dedicated care and keeping hospital records, Dr Simone Richardson for a critical review of the manuscript and the International AIDS Vaccine Initiative (IAVI) for facilitating EWN's collaboration. This manuscript is published with permission of the Director Kenya Medical Research Institute (KEMRI).

E.W.N., D.M.M., T.N., G.A. and P.L.M. contributed to the conception and design of the study, E.W.N., M.G., Y.S., T.H., D.Y. and I.O. performed the experiments, E.W.N. drafted the manuscript, D.M.M., M.G., T.N. and P.L.M. reviewed the manuscript.

E.W.N. and Y.S. are supported by the DELTAS Africa Initiative (DEL-15–003). The DELTAS Africa Initiative is an independent funding scheme of the African Academy of Sciences (AAS)'s Alliance for Accelerating Excellence in Science in Africa (AESA) and supported by the New Partnership for Africa's Development Planning and Coordinating Agency (NEPAD Agency) with funding from the Wellcome Trust (107769/Z/10/Z) and the UK government. The views expressed in this publication are those of the author(s) and not necessarily those of AAS, NEPAD Agency, Wellcome Trust or the UK government.

I.O., D.M.M. and T.N. are supported by the sub-Saharan African Network for TB/HIV Research Excellence (SANTHE), a DELTAS Africa Initiative (grant # DEL-15-006). The DELTAS Africa Initiative is an independent funding scheme of the African Academy of Sciences (AAS)'s Alliance for Accelerating Excellence in Science in Africa (AESA) and supported by the New Partnership for Africa's Development Planning and Coordinating Agency (NEPAD Agency) with funding from the Wellcome Trust (grant # 107752/Z/15/Z) and the UK government. The views expressed in this publication are those of the author(s) and not necessarily those of AAS, NEPAD Agency, Wellcome Trust or the UK government. M.G., D.Y. and G.A. are supported by the Ragon Institute of MGH, MIT and Harvard. T.N., (grant number 64809), T.H. and P.L.M. (grant number 98341) are supported by the South African Department of Science and Innovation through the National Research Foundation (South African Research Chairs Initiative).

Funding: this work was supported through a collaborative grant from the Sub-Saharan African Network for TB/HIV Research Excellence (SANTHE), a DELTAS Africa Initiative (grant # DEL-15-006). The DELTAS Africa Initiative is an independent funding scheme of the African Academy of Sciences (AAS)'s Alliance for Accelerating Excellence in Science in Africa (AESA) and supported by the New Partnership for Africa's Development Planning and Coordinating Agency (NEPAD Agency) with funding from the Wellcome Trust [grant # 107752/Z/15/Z] and the UK government. The views expressed in this publication are those of the author(s) and not necessarily those of AAS, NEPAD Agency, Wellcome Trust or the UK government.’

HIV antigens were kind donations from Drs Devin Sok, Joseph Jardine and Elise Landais, USA.

### Conflicts of interest

There are no conflicts of interest.

## Supplementary Material

Supplemental Digital Content

## Supplementary Material

Supplemental Digital Content

## Supplementary Material

Supplemental Digital Content

## Supplementary Material

Supplemental Digital Content

## Supplementary Material

Supplemental Digital Content

## References

[1] Moldt B Rakasz EG Schultz N Chan-Hui PY Swiderek K Weisgrau KL. Highly potent HIV-specific antibody neutralization in vitro translates into effective protection against mucosal SHIV challenge in vivo. *Proc Natl Acad Sci USA* 2012; 109:18921–18925. 23100539 10.1073/pnas.1214785109 PMC3503218

[2] Shingai M Donau OK Plishka RJ Buckler-White A Mascola JR Nabel GJ. Passive transfer of modest titers of potent and broadly neutralizing anti-HIV monoclonal antibodies block SHIV infection in macaques. *J Exp Med* 2014; 211:2061–2074. 25155019 10.1084/jem.20132494 PMC4172223

[3] Barouch DH Whitney JB Moldt B Klein F Oliveira TY Liu J. Therapeutic efficacy of potent neutralizing HIV-1-specific monoclonal antibodies in SHIV-infected rhesus monkeys. *Nature* 2013; 503:224–228. 24172905 10.1038/nature12744 PMC4017780

[4] Diskin R Klein F Horwitz JA Halper-Stromberg A Sather DN Marcovecchio PM. Restricting HIV-1 pathways for escape using rationally designed anti-HIV-1 antibodies. *J Exp Med* 2013; 210:1235–1249. 23712429 10.1084/jem.20130221 PMC3674693

[5] Klein F Halper-Stromberg A Horwitz JA Gruell H Scheid JF Bournazos S. HIV therapy by a combination of broadly neutralizing antibodies in humanized mice. *Nature* 2012; 492:118–122. 23103874 10.1038/nature11604 PMC3809838

[6] Caskey M Klein F Lorenzi JC Seaman MS West AP Jr Buckley N. Viraemia suppressed in HIV-1-infected humans by broadly neutralizing antibody 3BNC117. *Nature* 2015; 522:487–491. 25855300 10.1038/nature14411 PMC4890714

[7] Sather DN Armann J Ching LK Mavrantoni A Sellhorn G Caldwell Z. Factors associated with the development of cross-reactive neutralizing antibodies during human immunodeficiency virus type 1 infection. *J Virol* 2009; 83:757–769. 18987148 10.1128/JVI.02036-08 PMC2612355

[8] Stamatatos L. HIV vaccine design: the neutralizing antibody conundrum. *Curr Opin Immunol* 2012; 24:316–323. 22595693 10.1016/j.coi.2012.04.006

[9] Haynes BF Montefiori DC. Aiming to induce broadly reactive neutralizing antibody responses with HIV-1 vaccine candidates. *Exp Rev Vaccines* 2006; 5:579–595. 10.1586/14760584.5.4.579 16989638

[10] Goulder PJ Lewin SR Leitman EM. Paediatric HIV infection: the potential for cure. *Nat Rev Immunol* 2016; 16:259–271. 26972723 10.1038/nri.2016.19 PMC5694689

[11] Pereyra F Addo MM Kaufmann DE Liu Y Miura T Rathod A. Genetic and immunologic heterogeneity among persons who control HIV infection in the absence of therapy. *J Infect Dis* 2008; 197:563–571. 18275276 10.1086/526786

[12] Kiepiela P Leslie AJ Honeyborne I Ramduth D Thobakgale C Chetty S. Dominant influence of HLA-B in mediating the potential co-evolution of HIV and HLA. *Nature* 2004; 432:769–775. 15592417 10.1038/nature03113

[13] Simonich CA Williams KL Verkerke HP Williams JA Nduati R Lee KK. HIV-1 Neutralizing Antibodies with Limited Hypermutation from an Infant. *Cell* 2016; 166:77–87. 27345369 10.1016/j.cell.2016.05.055 PMC4930401

[14] Goo L Chohan V Nduati R Overbaugh J. Early development of broadly neutralizing antibodies in HIV-1-infected infants. *Nat Med* 2014; 20:655–658. 24859529 10.1038/nm.3565 PMC4060046

[15] Muenchhoff M Adland E Karimanzira O Crowther C Pace M Csala A. Nonprogressing HIV-infected children share fundamental immunological features of nonpathogenic SIV infection. *Sci Translat Med* 2016; 8:358ra125. 10.1126/scitranslmed.aag1048 PMC6087524 27683550

[16] Bournazos S DiLillo DJ Ravetch JV. The role of Fc-FcgammaR interactions in IgG-mediated microbial neutralization. *J Exp Med* 2015; 212:1361–1369. 26282878 10.1084/jem.20151267 PMC4548051

[17] Bournazos S Ravetch JV. Fcgamma receptor pathways during active and passive immunization. *Immunol Rev* 2015; 268:88–103. 26497515 10.1111/imr.12343 PMC7556827

[18] Nimmerjahn F Ravetch JV. Fcgamma receptors as regulators of immune responses. *Nat Rev Immunol* 2008; 8:34–47. 18064051 10.1038/nri2206

[19] Ackerman ME Dugast AS Alter G. Emerging concepts on the role of innate immunity in the prevention and control of HIV infection. *Annu Rev Med* 2012; 63:113–130. 22077718 10.1146/annurev-med-050310-085221

[20] Su B Dispinseri S Iannone V Zhang T Wu H Carapito R. Update on Fc-mediated antibody functions against HIV-1 beyond neutralization. *Front Immunol* 2019; 10:2968. 31921207 10.3389/fimmu.2019.02968 PMC6930241

[21] Ackerman ME Mikhailova A Brown EP Dowell KG Walker BD Bailey-Kellogg C. Polyfunctional HIV-specific antibody responses are associated with spontaneous HIV control. *PLoS Pathogens* 2016; 12:e1005315. 26745376 10.1371/journal.ppat.1005315 PMC4706315

[22] Corey L Gilbert PB Tomaras GD Haynes BF Pantaleo G Fauci AS. Immune correlates of vaccine protection against HIV-1 acquisition. *Sci Translat Med* 2015; 7:rv317. 10.1126/scitranslmed.aac7732 PMC4751141 26491081

[23] Bruel T Guivel-Benhassine F Amraoui S Malbec M Richard L Bourdic K. Elimination of HIV-1-infected cells by broadly neutralizing antibodies. *Nat Commun* 2016; 7:10844. 26936020 10.1038/ncomms10844 PMC4782064

[24] Halper-Stromberg A Lu CL Klein F Horwitz JA Bournazos S Nogueira L. Broadly neutralizing antibodies and viral inducers decrease rebound from HIV-1 latent reservoirs in humanized mice. *Cell* 2014; 158:989–999. 25131989 10.1016/j.cell.2014.07.043 PMC4163911

[25] Hessell AJ Hangartner L Hunter M Havenith CE Beurskens FJ Bakker JM. Fc receptor but not complement binding is important in antibody protection against HIV. *Nature* 2007; 449:101–104. 17805298 10.1038/nature06106

[26] Richardson SI Chung AW Natarajan H Mabvakure B Mkhize NN Garrett N. HIV-specific Fc effector function early in infection predicts the development of broadly neutralizing antibodies. *PLoS Pathog* 2018; 14:e1006987. 29630668 10.1371/journal.ppat.1006987 PMC5908199

[27] Muema DM Macharia GN Hassan AS Mwaringa SM Fegan GW Berkley JA. Control of viremia enables acquisition of resting memory b cells with age and normalization of activated B cell phenotypes in HIV-infected children. *J Immunol* 2015; 195:1082–1091. 26116511 10.4049/jimmunol.1500491 PMC4505960

[28] WHO. Antiretroviral therapy for HIV infection in infants and children. Recommendations for a public health approach 2010 revision. 2010.

[29] Blish CA Nedellec R Mandaliya K Mosier DE Overbaugh J. HIV-1 subtype A envelope variants from early in infection have variable sensitivity to neutralization and to inhibitors of viral entry. *Aids* 2007; 21:693–702. 17413690 10.1097/QAD.0b013e32805e8727

[30] Wei X Decker JM Liu H Zhang Z Arani RB Kilby JM. Emergence of resistant human immunodeficiency virus type 1 in patients receiving fusion inhibitor (T-20) monotherapy. *Antimicrob Agents Chemother* 2002; 46:1896–1905. 12019106 10.1128/AAC.46.6.1896-1905.2002 PMC127242

[31] Wei X Decker JM Wang S Hui H Kappes JC Wu X. Antibody neutralization and escape by HIV-1. *Nature* 2003; 422:307–312. 12646921 10.1038/nature01470

[32] Landais E Huang X Havenar-Daughton C Murrell B Price MA Wickramasinghe L. Broadly neutralizing antibody responses in a large longitudinal sub-Saharan HIV primary infection cohort. *PLoS Pathog* 2016; 12:e1005369. 26766578 10.1371/journal.ppat.1005369 PMC4713061

[33] Gray ES Madiga MC Hermanus T Moore PL Wibmer CK Tumba NL. The neutralization breadth of HIV-1 develops incrementally over four years and is associated with CD4+ T cell decline and high viral load during acute infection. *J Virol* 2011; 85:4828–4840. 21389135 10.1128/JVI.00198-11 PMC3126191

[34] Brown EP Licht AF Dugast AS Choi I Bailey-Kellogg C Alter G. High-throughput, multiplexed IgG subclassing of antigen-specific antibodies from clinical samples. *J Immunol Method* 2012; 386:117–123. 10.1016/j.jim.2012.09.007 PMC3475184 23023091

[35] McAndrew EG Dugast AS Licht AF Eusebio JR Alter G Ackerman ME. Determining the phagocytic activity of clinical antibody samples. *J Vis Exp* 2011; 57:e3588. 10.3791/3588 PMC3308623 22143444

[36] Ackerman ME Moldt B Wyatt RT Dugast AS McAndrew E Tsoukas S. A robust, high-throughput assay to determine the phagocytic activity of clinical antibody samples. *J Immunol Methods* 2011; 366:8–19. 21192942 10.1016/j.jim.2010.12.016 PMC3050993

[37] Chung AW Ghebremichael M Robinson H Brown E Choi I Lane S. Polyfunctional Fc-effector profiles mediated by IgG subclass selection distinguish RV144 and VAX003 vaccines. *Sci Translat Med* 2014; 6:228–238. 10.1126/scitranslmed.3007736 24648341

[38] Golay J Valgardsdottir R Musaraj G Giupponi D Spinelli O Introna M. Human neutrophils express low levels of FcgammaRIIIA, which plays a role in PMN activation. *Blood* 2019; 133:1395–1405. 30655272 10.1182/blood-2018-07-864538 PMC6484458

[39] Alter G Malenfant JM Delabre RM Burgett NC Yu XG Lichterfeld M. Increased natural killer cell activity in viremic HIV-1 infection. *J Immunol* 2004; 173:5305–5311. 15470077 10.4049/jimmunol.173.8.5305

[40] Moore PL. The neutralizing antibody response to the HIV-1 Env protein. *Curr HIV Res* 2018; 16:21–28. 29173180 10.2174/1570162X15666171124122044 PMC6234226

[41] Roider J Maehara T Ngoepe A Ramsuran D Muenchhoff M Adland E. High-frequency, functional HIV-specific T-follicular helper and regulatory cells are present within germinal centers in children but not adults. *Front Immunol* 2018; 9:1975. 30258437 10.3389/fimmu.2018.01975 PMC6143653

[42] Klasse J Blomberg J. Patterns of antibodies to human immunodeficiency virus proteins in different subclasses of IgG. *J Infect Dis* 1987; 156:1026–1030. 3479499 10.1093/infdis/156.6.1026

[43] Khalife J Guy B Capron M Kieny MP Ameisen JC Montagnier L. Isotypic restriction of the antibody response to human immunodeficiency virus. *AIDS Res Hum Retrovirus* 1988; 4:3–9. 10.1089/aid.1988.4.3 3163253

[44] Rowland-Jones S. Dimers are a girl's best friend. *Nat Med* 1997; 3:1199–1200. 9359691 10.1038/nm1197-1199

[45] Tomaras GD Haynes BF. HIV-1-specific antibody responses during acute and chronic HIV-1 infection. *Curr Opin HIV AIDS* 2009; 4:373–379. 20048700 10.1097/COH.0b013e32832f00c0 PMC3133462

[46] Tjiam MC Taylor JP Morshidi MA Sariputra L Burrows S Martin JN. Viremic HIV controllers exhibit high plasmacytoid dendritic cell-reactive opsonophagocytic IgG antibody responses against HIV-1 p24 associated with greater antibody isotype diversification. *J Immunol* 2015; 194:5320–5328. 25911748 10.4049/jimmunol.1402918 PMC4492937

[47] French MA Abudulai LN Fernandez S. Isotype diversification of IgG antibodies to HIV Gag proteins as a therapeutic vaccination strategy for HIV infection. *Vaccines (Basel)* 2013; 1:328–342. 26344116 10.3390/vaccines1030328 PMC4494226

[48] Tomaras GD Yates NL Liu P Qin L Fouda GG Chavez LL. Initial B-cell responses to transmitted human immunodeficiency virus type 1: virion-binding immunoglobulin M (IgM) and IgG antibodies followed by plasma antigp41 antibodies with ineffective control of initial viremia. *J Virol* 2008; 82:12449–12463. 18842730 10.1128/JVI.01708-08 PMC2593361

[49] E-B Rossheim A Cunningham TD Hair PS Shah T Cunnion KN Troy SB. Effects of well controlled HIV infection on complement activation and function. *J Acquir Immune Defic Syndr* 2016; 73:20–26. 27192377 10.1097/QAI.0000000000001079 PMC4981513

[50] Carroll MC Isenman DE. Regulation of humoral immunity by complement. *Immunity* 2012; 37:199–207. 22921118 10.1016/j.immuni.2012.08.002 PMC5784422

[51] Fang Y Xu C Fu YX Holers VM Molina H. Expression of complement receptors 1 and 2 on follicular dendritic cells is necessary for the generation of a strong antigen-specific IgG response. *J Immunol* 1998; 160:5273–5279. 9605124

[52] Chung AW Crispin M Pritchard L Robinson H Gorny MK Yu X. Identification of antibody glycosylation structures that predict monoclonal antibody Fc-effector function. *AIDS* 2014; 28:2523–2530. 25160934 10.1097/QAD.0000000000000444 PMC4429604

[53] Ljunggren K Broliden PA Morfeldt-Manson L Jondal M Wahren B. IgG subclass response to HIV in relation to antibody-dependent cellular cytotoxicity at different clinical stages. *Clin Exp Immunol* 1988; 73:343–347. 3208446 PMC1541760

[54] Binley JM Lybarger EA Crooks ET Seaman MS Gray E Davis KL. Profiling the specificity of neutralizing antibodies in a large panel of plasmas from patients chronically infected with human immunodeficiency virus type 1 subtypes B and C. *J Virol* 2008; 82:11651–11668. 18815292 10.1128/JVI.01762-08 PMC2583680

[55] Hristodorov D Fischer R Linden L. With or without sugar? (A)glycosylation of therapeutic antibodies. *Mol Biotechnol* 2013; 54:1056–1068. 23097175 10.1007/s12033-012-9612-x

[56] Chung AW Kumar MP Arnold KB Yu WH Schoen MK Dunphy LJ. Dissecting polyclonal vaccine-induced humoral immunity against HIV using systems serology. *Cell* 2015; 163:988–998. 26544943 10.1016/j.cell.2015.10.027 PMC5490491

[57] Worley MJ Fei K Lopez-Denman AJ Kelleher AD Kent SJ Chung AW. Neutrophils mediate HIV-specific antibody-dependent phagocytosis and ADCC. *J Immunol Methods* 2018; 457:41–52. 29605231 10.1016/j.jim.2018.03.007

[58] Chung AW Rollman E Center RJ Kent SJ Stratov I. Rapid degranulation of NK cells following activation by HIV-specific antibodies. *J Immunol* 2009; 182:1202–1210. 19124764 10.4049/jimmunol.182.2.1202

[59] Perez LG Costa MR Todd CA Haynes BF Montefiori DC. Utilization of immunoglobulin G Fc receptors by human immunodeficiency virus type 1: a specific role for antibodies against the membrane-proximal external region of gp41. *J Virol* 2009; 83:7397–7410. 19458010 10.1128/JVI.00656-09 PMC2708617

[60] Kinoshita K Honjo T. Linking class-switch recombination with somatic hypermutation. *Nat Rev Mol Cell Biol* 2001; 2:493–503. 11433363 10.1038/35080033

[61] Janda A Bowen A Greenspan NS Casadevall A. Ig constant region effects on variable region structure and function. *Front Microbiol* 2016; 7:22. 26870003 10.3389/fmicb.2016.00022 PMC4740385

