# Supplementary figures and images for: Coordinated Fc-effector and neutralization functions in HIV-infected children define a window of opportunity for HIV vaccination
Source: AIDS. 2021 Jun 10;35(12):1895–905. doi: 10.1097/QAD.0000000000002976 (PMC8462450; doi:10.1097/QAD.0000000000002976)

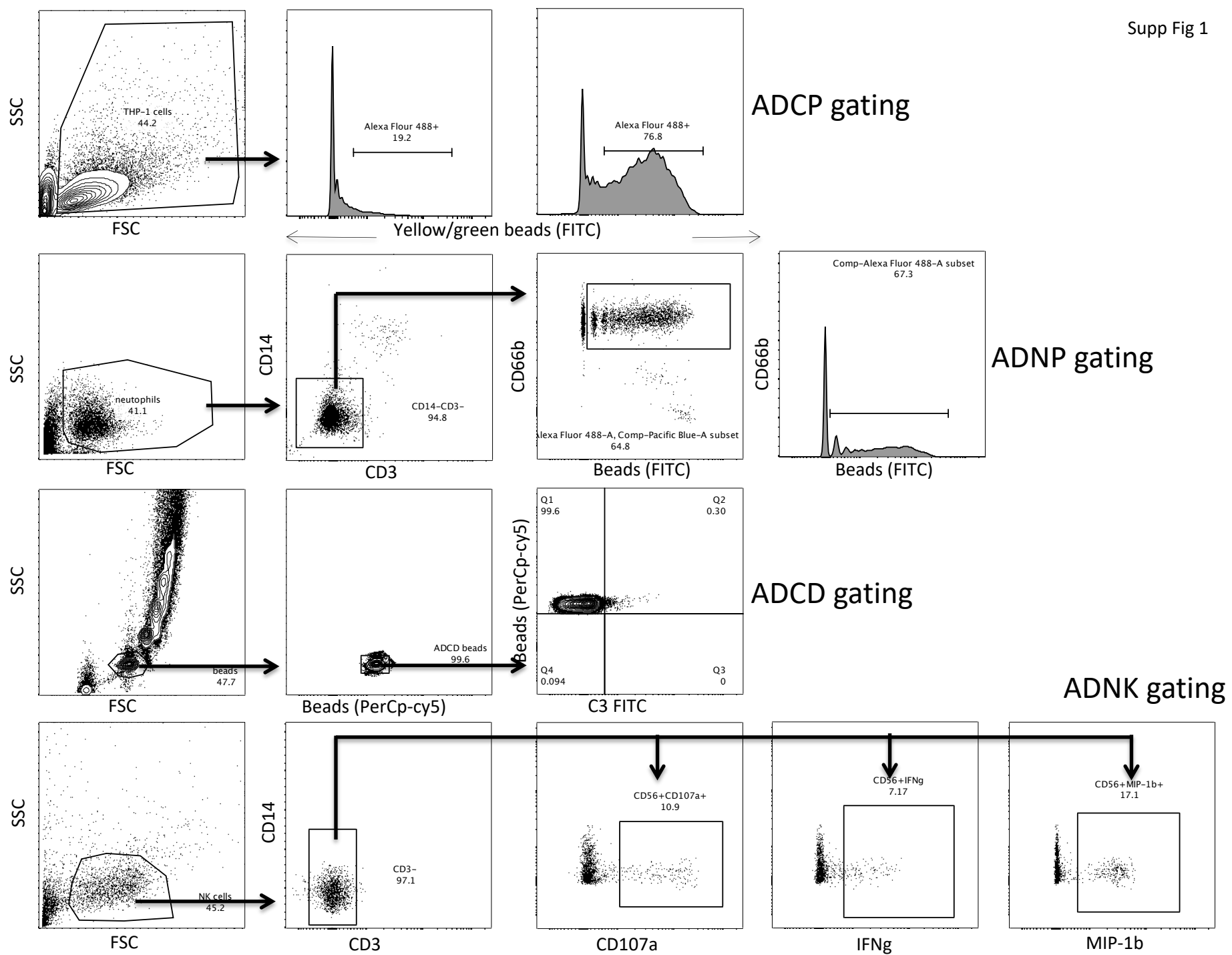

Supplement: Supplemental Digital Content [file aids-35-1895-s001.pdf]

a)

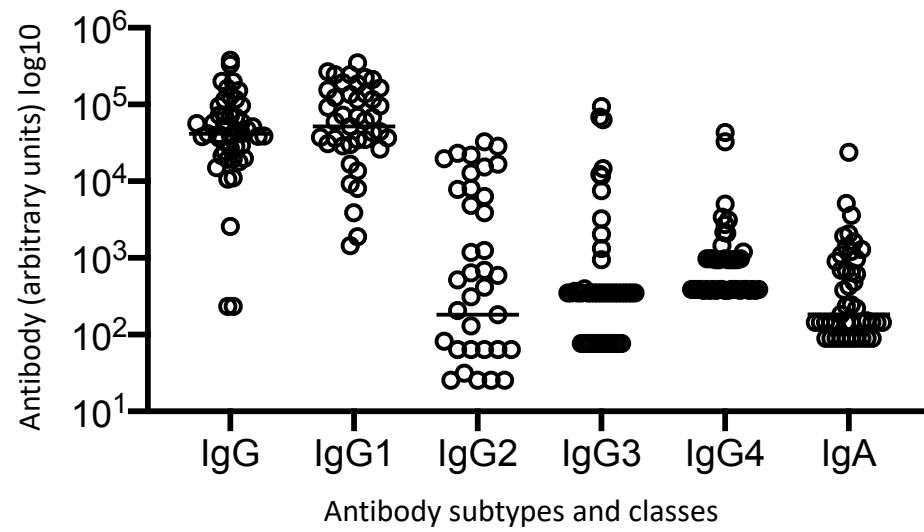

b)

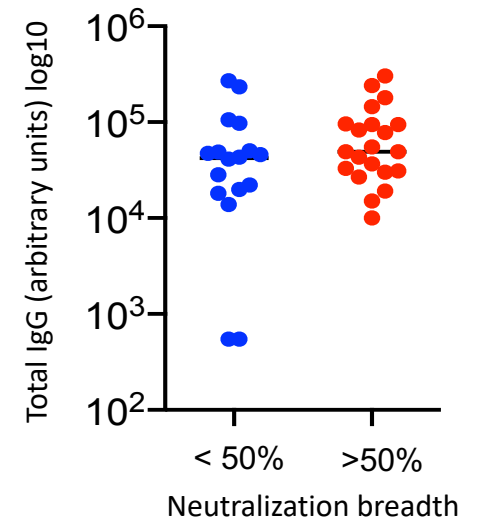

Supplement: Supplemental Digital Content [file aids-35-1895-s002.pdf]

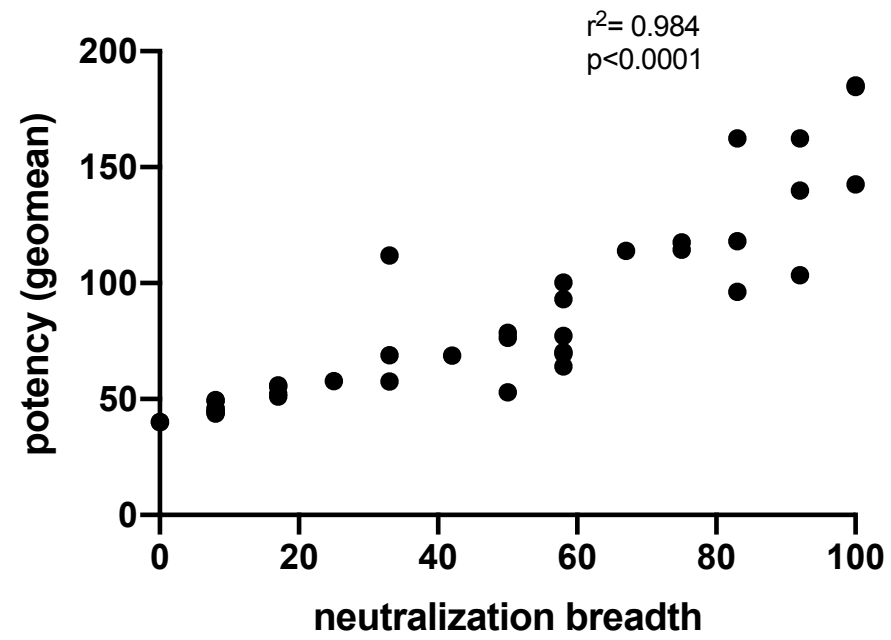

Supplement: Supplemental Digital Content [file aids-35-1895-s003.pdf]

a

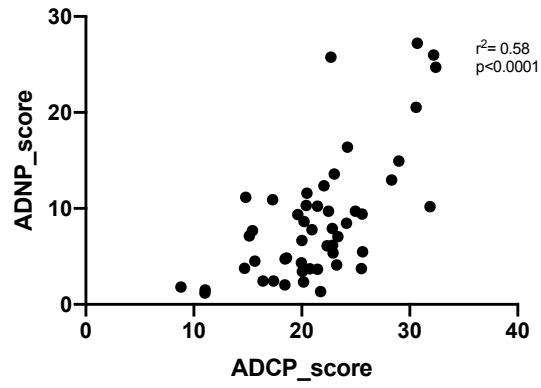

b

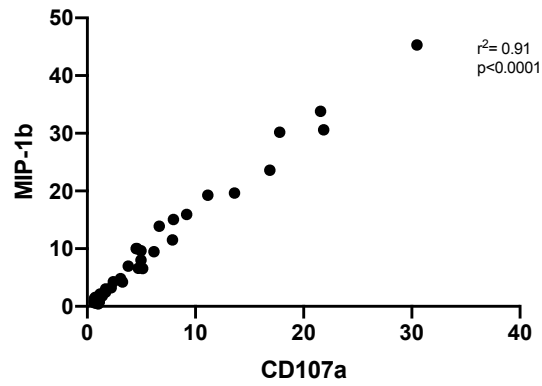

c

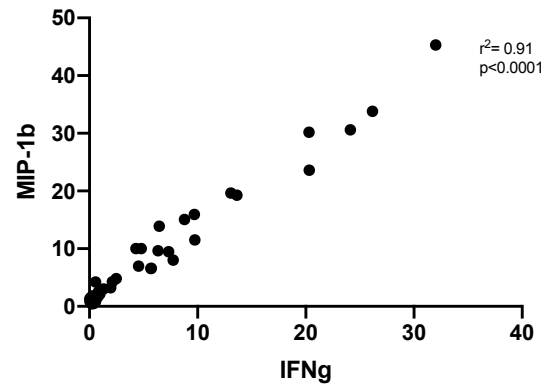

d

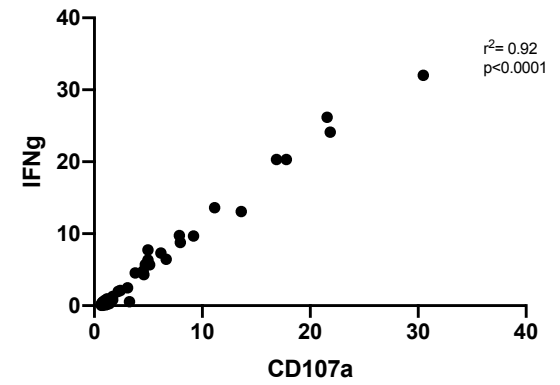

Supplement: Supplemental Digital Content [file aids-35-1895-s004.pdf]
