# Supplementary material for: Coordinated Fc-effector and neutralization functions in HIV-infected children define a window of opportunity for HIV vaccination
Source: AIDS. 2021 Jun 10;35(12):1895–905. doi: 10.1097/QAD.0000000000002976 (PMC8462450; doi:10.1097/QAD.0000000000002976)
Supplement: Supplemental Digital Content [file aids-35-1895-s005.pdf]

Spearman’s rho

	AGE	IgG	IgG1	IgG2	IgG3	IgG4	IgA	IgA1	IgA2	ADCP	ADNP	ADCD	IFNg	MIP-1b	CD107a	CD4 PER	CD4 COUNT	VIRAL LOAD	NEUT BREADTH	FC POLYFUNCTION
AGE	1	0.04779	0.276304	0.324925	0.147867	0.44639	0.467108	0.513397	0.321962	0.157551	0.410187	0.25224	0.304526	0.412689	0.413293	-0.091915	-0.400226604	0.089725964	0.376611071	0.316684249
IgG	0.04779	1	0.818347	0.442914	0.512175	0.405022	0.346525	0.28449	0.531813	0.398569	0.531089	0.520474	0.434255	0.471428	0.417461	-0.33917	-0.255230776	0.097992879	0.395371219	0.576911089
IgG1	0.276304	0.818347	1	0.573781	0.520553	0.477866	0.488354	0.42859	0.672332	0.441502	0.712516	0.671805	0.463989	0.597604	0.53277	-0.37753	-0.358161351	0.052582183	0.545631028	0.661660079
IgG2	0.324925	0.442914	0.573781	1	0.3	0.288933	0.460001	0.522793	0.479051	0.493017	0.458893	0.523452	0.354898	0.44919	0.431994	-0.4917	-0.476172608	0.286009516	0.81144182	0.527009223
IgG3	0.147867	0.512175	0.520553	0.3	1	0.632279	0.351872	0.334519	0.357971	0.220422	0.348617	0.378524	0.217054	0.257928	0.236082	-0.306478	-0.298311445	0.312112814	0.223413786	0.316996047
IgG4	0.44639	0.405022	0.477866	0.288933	0.632279	1	0.445423	0.500264	0.510145	0.17365	0.347299	0.422003	0.301621	0.396195	0.313319	-0.051822	-0.290056285	0.280563504	0.489224577	0.30342556
IgA	0.467108	0.346525	0.488354	0.460001	0.351872	0.445423	1	0.772084	0.522525	0.509674	0.549045	0.462448	0.472233	0.6063	0.57861	-0.271691	-0.394558316	0.172459498	0.462073216	0.631298852
IgA1	0.513397	0.28449	0.42859	0.522793	0.334519	0.500264	0.772084	1	0.711462	0.461792	0.486166	0.468116	0.533051	0.634531	0.537844	-0.336032	-0.419699812	0.301971964	0.57217524	0.576679842
IgA2	0.321962	0.531813	0.672332	0.479051	0.357971	0.510145	0.522525	0.711462	1	0.390119	0.445718	0.547431	0.453982	0.575758	0.486258	-0.315587	-0.327204503	0.153615091	0.614019241	0.523188406
ADCP	0.157551	0.398569	0.441502	0.493017	0.220422	0.17365	0.509674	0.461792	0.390119	1	0.581561	0.666267	0.528265	0.633304	0.589123	-0.232315	-0.152219873	0.167983117	0.403633404	0.814453782
ADNP	0.410187	0.531089	0.712516	0.458893	0.348617	0.347299	0.549045	0.486166	0.445718	0.581561	1	0.610084	0.630408	0.745115	0.664568	-0.426789	-0.458169737	0.166572679	0.45258221	0.844993998
ADCD	0.25224	0.520474	0.671805	0.523452	0.378524	0.422003	0.462448	0.468116	0.547431	0.666267	0.610084	1	0.711327	0.822188	0.790382	-0.304108	-0.288734521	0.094499318	0.442352168	0.779207683
IFNg	0.304526	0.434255	0.463989	0.354898	0.217054	0.301621	0.472233	0.533051	0.453982	0.528265	0.630408	0.711327	1	0.909032	0.92119	-0.258014	-0.268454744	0.074051706	0.554361529	0.814795918
MIP-1b	0.412689	0.471428	0.597604	0.44919	0.257928	0.396195	0.6063	0.634531	0.575758	0.633304	0.745115	0.822188	0.909032	1	0.907729	-0.328518	-0.412020906	0.117184371	0.576178685	0.90588363
CD107a	0.413293	0.417461	0.53277	0.431994	0.236082	0.313319	0.57861	0.537844	0.486258	0.589123	0.664568	0.790382	0.92119	0.907729	1	-0.234334	-0.278397213	0.021977138	0.572644789	0.860508033
CD4 PER	-0.091915	-0.33917	-0.37753	-0.4917	-0.306478	-0.051822	-0.271691	-0.336032	-0.315587	-0.232315	-0.426789	-0.304108	-0.258014	-0.328518	-0.234334	1	0.767117738	-0.471839761	-0.345405445	-0.388380196
CD4 COUNT	-0.400227	-0.255231	-0.358161	-0.476173	-0.298311	-0.290056	-0.394558	-0.4197	-0.327205	-0.15222	-0.45817	-0.288735	-0.268455	-0.412021	-0.278397	0.767118	1	-0.471575859	-0.466328254	-0.382059801
VIRAL LOAD	0.089726	0.097993	0.052582	0.28601	0.312113	0.280564	0.172459	0.301972	0.153615	0.167983	0.166573	0.094499	0.074052	0.117184	0.021977	-0.47184	-0.471575859	1	0.150877128	0.137094533
NEUT BREADTH	0.376611	0.395371	0.545631	0.811442	0.223414	0.489225	0.462073	0.572175	0.614019	0.403633	0.452582	0.442352	0.554362	0.576179	0.572645	-0.345405	-0.466328254	0.150877128	1	0.597356719
FC POLYFUNCTION	0.316684	0.576911	0.66166	0.527009	0.316996	0.303426	0.631299	0.57668	0.523188	0.814454	0.844994	0.779208	0.814796	0.905884	0.860508	-0.38838	-0.382059801	0.137094533	0.597356719	1

p values

	AGE	IgG	IgG1	IgG2	IgG3	IgG4	IgA	IgA1	IgA2	ADCP	ADNP	ADCD	IFNg	MIP-1b	CD107a	CD4 PER	CD4 COUNT	VIRAL LOAD	NEUT BREADTH	FC POLYFUNCTION
AGE		0.755236	0.08034	0.03819	0.356206	0.003439	0.001217	0.000597	0.040089	0.301314	0.005134	0.0946	0.044446	0.005956	0.005876	0.562637986	0.007830377	0.56244686	0.025749527	0.034048973
IgG	0.755236		6.64E-12	0.002312	0.000322	0.005782	0.013692	0.058217	0.00017	0.004146	7.25E-05	0.000107	0.001823	0.000719	0.003156	0.027997069	0.098569671	0.526848326	0.017001971	1.15795E-05
IgG1	0.08034	6.64E-12		3.78E-05	0.000247	0.000901	0.000666	0.00331	4.24E-07	0.002397	4.02E-08	4.36E-07	0.001512	1.84E-05	0.000197	0.017817064	0.023253258	0.747273527	0.001238247	7.46709E-07
IgG2	0.03819	0.002312	3.78E-05		0.045262	0.054235	0.001477	0.000229	0.000871	0.00058	0.001521	0.000224	0.018085	0.002223	0.003409	0.001477753	0.001897258	0.073597832	1.76099E-08	0.000199741
IgG3	0.356206	0.000322	0.000247	0.045262		3.18E-06	0.017771	0.024703	0.015762	0.145673	0.018928	0.010349	0.156991	0.090955	0.122878	0.057744844	0.061527895	0.049917742	0.219017197	0.033863552
IgG4	0.003439	0.005782	0.000901	0.054235	3.18E-06		0.002168	0.000466	0.000344	0.253958	0.019415	0.003884	0.04662	0.00776	0.038363	0.754046181	0.069438041	0.079501754	0.004489056	0.042743314
IgA	0.001217	0.013692	0.000666	0.001477	0.017771	0.002168		5.29E-10	0.000231	0.000157	3.65E-05	0.000722	0.000613	4.94E-06	1.65E-05	0.081765866	0.008838884	0.262949556	0.004552279	8.86219E-07
IgA1	0.000597	0.058217	0.00331	0.000229	0.024703	0.000466	5.29E-10		4.3E-08	0.001407	0.00071	0.001184	0.000195	3.74E-06	0.000166	0.036483742	0.007017948	0.058257841	0.000622531	3.38042E-05
IgA2	0.040089	0.00017	4.24E-07	0.000871	0.015762	0.000344	0.000231	4.3E-08		0.008067	0.002152	9.95E-05	0.001966	4.33E-05	0.000818	0.050341059	0.039311068	0.343958529	0.000185564	0.00022644
ADCP	0.301314	0.004146	0.002397	0.00058	0.145673	0.253958	0.000157	0.001407	0.008067		9.46E-06	1.28E-07	9.57E-05	1.36E-06	1.06E-05	0.138745748	0.329831524	0.275729077	0.014638437	6.26091E-13
ADNP	0.005134	7.25E-05	4.02E-08	0.001521	0.018928	0.019415	3.65E-05	0.00071	0.002152	9.46E-06		2.56E-06	1.21E-06	1.25E-09	2.59E-07	0.004822658	0.002004221	0.27983888	0.00557976	1.20513E-14
ADCD	0.0946	0.000107	4.36E-07	0.000224	0.010349	0.003884	0.000722	0.001184	9.95E-05	1.28E-07	2.56E-06		1.01E-08	7.79E-13	2.38E-11	0.050227554	0.060411186	0.541755914	0.006905096	2.64852E-11
IFNg	0.044446	0.001823	0.001512	0.018085	0.156991	0.04662	0.000613	0.000195	0.001966	9.57E-05	1.21E-06	1.01E-08		4.18E-19	1.76E-20	0.103373619	0.085615375	0.636977134	0.000549284	1.04807E-12
MIP-1b	0.005956	0.000719	1.84E-05	0.002223	0.090955	0.00776	4.94E-06	3.74E-06	4.33E-05	1.36E-06	1.25E-09	7.79E-13	4.18E-19		5.71E-19	0.03848244	0.007435381	0.459861624	0.0003624	8.82445E-19
CD107a	0.005876	0.003156	0.000197	0.003409	0.122878	0.038363	1.65E-05	0.000166	0.000818	1.06E-05	2.59E-07	2.38E-11	1.76E-20	5.71E-19		0.145554356	0.077982467	0.890124699	0.000401406	4.53404E-15
CD4 PER	0.562638	0.027997	0.017817	0.001478	0.057745	0.754046	0.081766	0.036484	0.050341	0.138746	0.004823	0.050228	0.103374	0.038482	0.145554		3.12181E-09	0.001843293	0.048980281	0.011032068
CD4 COUNT	0.00783	0.09857	0.023253	0.001897	0.061528	0.069438	0.008839	0.007018	0.039311	0.329832	0.002004	0.060411	0.085615	0.007435	0.077982	3.12181E-09		0.001618526	0.005438061	0.011463828
VIRAL LOAD	0.562447	0.526848	0.747274	0.073598	0.049918	0.079502	0.26295	0.058258	0.343959	0.275729	0.279839	0.541756	0.636977	0.459862	0.890125	0.001843293	0.001618526		0.386955267	0.374861993
NEUT BREADTH	0.02575	0.017002	0.001238	1.76E-08	0.219017	0.004489	0.004552	0.000623	0.000186	0.014638	0.00558	0.006905	0.000549	0.000362	0.000401	0.048980281	0.005438061	0.386955267		0.000119846
FC POLYFUNCTION	0.034049	1.16E-05	7.47E-07	0.0002	0.033864	0.042743	8.86E-07	3.38E-05	0.000226	6.26E-13	1.21E-14	2.65E-11	1.05E-12	8.82E-19	4.53E-15	0.011032068	0.011463828	0.374861993	0.000119846	
